# Forced Respiration in Opium Poisoning—Its Possibilities, and the Apparatus Best Adapted to Produce It

**Published:** 1887-11

**Authors:** Geo. E. Fell

**Affiliations:** Professor of Physiology and Microscopy, Medical Department of Niagara University, Fellow of the New York State Medical Association, President of the Buffalo Microscopical Club, Physician to Sisters of Charity Hospital, etc., Buffalo, N. Y.


THE BUFFALO Medical AND Surgical Journal

VOL. XXVIII. NOVEMBER, 1887. NO. 4

### Original Communications.

### *FORCED RESPIRATION IN OPIUM POISONING—ITS POSSIBILITIES, AND THE APPARATUS BEST ADAPTED TO PRODUCE IT.**

By GEO. E. FELL, M. D., F. R. M. S.,

Professor of Physiology and Microscopy, Medical Department of Niagara University, Fellow of the New York State Medical Association, President of the Buffalo Microscopical Club, Physician to Sisters of Charity Hospital, etc., Buffalo, N. Y.

In one of the latest extensive medical works†, we find that artificial respiration has a wider range of application than might at first be supposed. It is said to have been used in "drowning, strangulation, occlusion of the air passages, poisoning by carbonic acid gas, chloroform, opium or its alkaloids, strychnia, woorara, snake-bite, in hemorrhage, or even in nervous shock from a blow or fall." Of the methods, a few may be mentioned, as those of "Marshall Hall," "Sylvester," "Howard," and others, which depend for their success upon the movements of the limbs or body of the patient, supplemented, usually, by pressure of the thorax on the part of the physician; those in which tubes and mouth-pieces are used, and the air supplied by bellows of different forms, and the methods used on various animals in the physiological laboratories, in which the trachea is opened to supply the air for respiration.

* Read before the Section of General Medicine of the International Medical Congress, Washington, D. C., Sept. 9, 1887, and before the Buffalo Medical Union, Oct. 26, 1887.

† Wood's Reference Hand-Book of the Medical Sciences.

The first of this series of methods is that generally adopted, and may be found efficacious so long as the muscles of respiration retain their tonicity, and the heart has not ceased to beat.

The second series, in which mouth-tubes are used, may prove uncertain in action, owing to the difficulty of passing the air into the (paralysed) larynx, with the æsophagus presenting a more direct road for its passage to the stomach.

The third series, differing from the others, presents a *positive* method. By opening into the trachea, we are enabled to force air into the lungs, and by forcible pressure on the thorax, if needed, expel it.

To the latter method, I suggest the term *forced*, to distinguish it from the ordinary *artificial respiration.*

After extensive reference to the most important medical works, and with the assistance of some of the leading practitioners of Buffalo, I have been unable to find a case in which *forced respiration* has been used upon a human subject in opium poisoning. The question may be raised by some of our ultra-conservative practitioners as to whether this operation of tracheotomy, with its supposed attendant dangers, and application of forced respiration, even in extreme cases, is justifiable? Or would not the ordinary methods of artificial respiration usually employed be quite sufficient? I am pleased to answer this question, by recounting the case which influenced and determined me to make the operation when opportunity offered.

### CASE OF THOS. J. DYKE.

Thos. J. Dyke, a night clerk in Mr. C. M. Lyman's drug store, Buffalo, took an over-dose of morphine about midnight, June 22, 1886. On the morning of the 23d inst., as soon as he was discovered, some other physicians, with myself, were called to attend him.

The symptomatic effects of the poison were markedly evidenced, and with the whole armamentarium of a most complete drug store at our disposal, it was hoped we might save our patient. Electricity, atropia, coffee extract, brandy and active movements were used. *Artificial respiration*, by "Sylvester's" method, was also employed; for a time it appeared to be beneficial, but only for a short time, as the patient soon succumbed to the inevitable. At this time, I felt keenly the inadequacy of the methods at our command, and then resolved, if opportunity ever offered, to make the operation which I have now to record, and which, I believe, might have saved Mr. Dyke's life.

In my opinion, after artificial respiration fails in cases of this nature, the *forced respiration* indicated is justifiable, and the value of its application will, I feel sure, be admitted, after the recital of the case which I now have the honor of presenting to you.

### CASE OF PATRICK BURNS.

At 12.30 A. M., Saturday, July 23, 1887, I was hurriedly called to attend Mr. Patrick Burns, book-keeper, residing at No. 49 Morgan street. I found the patient in a semi-conscious condition. His wife reported he had been drinking heavily for a week past, and had been in the habit of using alcoholic liquors to excess for ten or twelve years.

His present excesses had induced him to try chloral to produce sleep, but not finding this to succeed, he had added morphine, with the results recorded in the following pages. According to his statement, he had taken the drug late Friday afternoon, so that sufficient time had elapsed to permit complete absorption. When first discovered by his wife, he was breathing sturturously, and was with difficulty aroused. A draught of black coffee was given, which produced vomiting. On my arrival, I supplemented this with one of mustard, sodium chloride and water, which effectually emptied the stomach. This produced no further effect, as the patient, left to himself, immediately passed into the deep, narcotic condition of opium poisoning. The pupils were markedly contracted, and it was evident a serious case was in hand. At this time, I administered two cathartic compound pills which I had with me, and, at different times, minim doses of fluid extract of belladonna, sent for some atropia, and frequently administered the one-sixtieth of a grain hypodermically. To keep the patient awake, he was dressed and walked around the block in the cool, pure atmosphere of the early morning. At each round I examined him and administered more atropia. The fourth or fifth round, when within one-half block of the house, his limbs gave out, and while being tugged and jerked along, sturturous breathing began again; he was carried into the house and laid on the floor, as I believed, to die. This was about 3.30 A. M. As the respirations failed, and the intervals between them lengthened, *Sylvester's method of artificial respiration was employed, and kept up at intervals* long after I had given up any hopes of the man's recovery and until I was thoroughly exhausted, and, *further*, without apparent benefit to the patient. In the meantime, I notified the family that the patient could not live, with the effect of producing the scenes usually associated with tragic death in the household; Mrs. Burns had a slight attack of hysteria, requiring my attention for a time. At this juncture, Father Grant, of the Cathedral, appeared, and performed the last rites of the Catholic Church. At my suggestion, a bed was prepared in the front parlor of the house, and the patient laid upon it. From Mrs. Burns, I obtained the data for the death certificate, which I confidently expected to file in the morning. I then took a last look at the patient, only to confirm my opinion that death was imminent.

The pulse, before Father Grant came, had registered as high as 180, and before I left the house it could have been counted with difficulty—I considered it 200 or more. The respirations at four o'clock in the morning were five per minute, and, when I left the house for home, were intermittent, or with a long intermission followed by a few spasmodic respiratory efforts, and then apparent inanition for a time. I may be criticised for leaving my patient at this time, but I was tired out (no excuse), and fully believed no power on earth could save him; in fact, informed his wife that I did not think calling another physician would do any good.

With an unaccountable reluctancy, I left for home a little after five o'clock in the morning, went to bed, and, after a sound sleep, was awakened by a call about eight o'clock.

Dr. F. R. Campbell, Professor of Materia Medica, Medical Department, Niagara University, who, through illness, had been unable to respond to an early summons from Mrs. Burns, called about eight A. M., and finding Mr. Burns still alive, sent for me. I promptly repaired to the house, and, indeed, found the patient alive, with respirations *not more* than one per minute, and the pulse with difficulty to be detected at the wrist.

The extremities were quite cold at this time, the face had assumed a cyanotic appearance; pupils were still contracted. The doctor suggested that more atropiæ be given hypodermically, to which I assented. Together, we repaired to the drug store near by, had some powders prepared, and on our return were surprised to find the pupils *widely dilated;* it is needless to say *no more* atropia was administered. The sudden dilatation of the pupils was, undoubtedly, caused by the paralysis of the nerve centers controlling the iris, and is one of the frequent conditions in the *last stages* of opium poisoning, and indicative of general muscular paralysis; it is also known as the "dilatation of asphyxia."

Dr. Campbell made the remark: "We can do nothing more now." I agreed with him, but recalling the case of Mr. Dyke, and having had a number of similar cases, some of which had recovered and others proved fatal, when the ordinary methods of artificial respiration had proved unavailing, I had frequently thought if the respirations could be kept up by suitable means for a sufficient time to permit the elimination of the poison, life might be saved. Having held the chair of Physiology and Microscopy in the Medical Department of Niagara University since the institution of the department, I was conversant with the procedure and requirements in the performance of artificial respirations on dogs, and believed the method used in my laboratory could just as well be performed on the human subject. This, coupled with the fact that I am engaged in the active practice of medicine, seemed to offer the conditions on the part of the physician, by which the operation I am about to describe was brought about to be made of practical value in the saving of human life. I informed Dr. Campbell that Mr. Burns' life could, in my opinion, only be saved by opening the trachea, placing a tube in it, and, with suitable apparatus, keep up the respirations until the poison could be eliminated. I informed him that I had the apparatus used on dogs in the laboratory of the college at my residence near by. He, to my surprise, offered to assist if I would make the experiment. As expeditiously as possible, with the aid of a gentleman stopping at the house, I obtained the apparatus I exhibit here, and, with the exception of having the tracheal tube covered with dog-hair and blood*, it is in the same condition as when used on Mr. Burns. On my way, I asked Dr. G. H. McMichael† to assist in the operation.

### DETAILS OF OPERATION.

The tracheal tube was quickly cleaned with a bi-chloride solution, and, with instruments illy adapted to the purpose, the operation was begun at nine o'clock A. M. As tracheotomy cannot offer any points of interest, reference need not be made to it. After making the initial low incision, Dr. Campbell suggested that *laryngotomy* would take less time; I hesitated, but thinking that Mr. Burns' voice would be in better condition *after recovery*, on account of less danger to the vocal chords, with the low operation, I continued the opening.

The hemorrhage, which was considerable, was soon overcome with hæmostatic forceps and ligation. Before incising the trachea, it was reduced to a minimum. The greatest difficulty was experienced in passing a ligature about the trachea to prevent the air from passing up the throat. After this was accomplished, we were ready to begin the respirations. The color of the blood passing from the incision was of a *dark* coffee-color, indicating an extreme venous condition.

---

*A few days before this, the apparatus was used in electrical experiments on a dog at the dog-pound.

† Dr. McMichael's niece, a little girl, hearing about the proposed operation, informed her cousin, a reporter on the *News*, that the doctors were operating on a man's throat. Before the operation was completed, this reporter appeared upon the scene, vanished, and, before I had left the bedside, the operation was being heralded about the streets. As criticism has been made in the pages of this JOURNAL, deploring the press publication, I deem it a duty to show I was wholly irresponsible for its appearance. When I was subsequently interviewed, knowing full well I could no more keep this case from the press, after having once appeared, than I could keep the sun from rising, I gave merely a plain account, and am not responsible for the reportorial "gush" accompanying the criticised article.—G. E. F.

Having been deeply occupied with the operation, I had not noticed the condition of the patient further than to state that no respiratory effort had been made for some time, and the dark-blue tinge to the face had materially increased.

We began the *forced respirations*, and, as some interesting physiological changes ensued, it may be well to note them carefully. The lungs were inflated; not the slightest expiratory effort was made indicating paralyzation of not only the muscles of respiration, but the loss of elasticity in the lung tissue.*

Physiological experiments have demonstrated that the heart in the lower animals, shortly after ceasing to beat, may again be called into action by passing arterial blood through its channels.

We may readily imagine the human organism in asphyxia, or where the heart is weakened by insufficient nourishment, in a condition similating this, and where, by re-supplying the heart with oxygenated blood, its life may be retained. Who will say that a human heart, after ceasing to beat rythmically, may not yet be called into action? And are not the conditions for re-supplying the weakened heart with nourishment provided in forced respiration, and its practical value demonstrated in this case of Mr. Burns?

We stated that no expiratory effort had been made on the part of the patient; we had forced the inspiration, and now forced expiration was effected in the best manner by pressure on the thorax.

In forced expiration, we must necessarily send the blood in the capillaries of the lungs, which, by our forced inspiration, has been oxydised, into the left cavities of the heart, where not only does it nourish the waning heart muscle, but causes, to a certain degree, expansion of the left auricle, and produces a tendency to return to its rythmical action.

### CHANGES OBSERVED IN PATIENT.

The effect of the forced respirations were observed much sooner *on the heart* than on *the lungs*. The change from the venous to a more arterial state of the blood was the one first

* Are not these the conditions calling for *forced respiration*, and where artificial respiration would certainly fail?

noticed; it was also observed that the pulse at the wrist had become stronger and quite regular; this was some time before any attempts at respiration on the part of the patient were noticed. The forced respirations, in fact, were kept up some twenty to thirty minutes before any attempts at breathing were noted, and, before others were observed, a long interval elapsed; in the meantime, the face had begun to assume a more natural appearance, the dark, venous color had given way to the natural ruddy appearance of health, and then came two or three quick, hurried movements of the lungs, the *first attempts* at natural breathing. The lungs subsided, and without both *forced* inspirations and expirations, some time after this, our patient would have quickly succumbed. Sensibility was quickly returning, as evidenced by movements of the muscles of the eye when pressed by the finger.

The next feature of interest was a movement of the limbs, quickly followed by one of the arms, and, shortly following this, the eyes opened, staring wildly. We soon found that our patient could hear and understand. Water was offered and eagerly drank; inclinations or slight nods of the head would indicate when he desired more and had had enough. All this time, we were performing the respiratory acts for him, and to me the various effects produced by *withholding* or supplying an *excess* of air were extremely interesting, and enhanced from the fact that it was a human being we were operating upon. Without knowing what the future of the case might develop, I believed at this time I had saved this man's life, and that he would fully recover.

After about two hours' work, the natural respirations became longer and longer, until they appeared almost normal. The opening in the side of the valve (one-eighth by one-half inch, or one and four-tenths by three-tenths mm.), which permitted the natural respirations, was not large enough to supply the lungs with their full requisite of air, so that natural breathing could only be carried on for a short time before dyspnœa would ensue. This was evidenced on the part of the patient by gasps and uneasy movements. When this occurred, the natural were supplemented by the *forced respirations*, with the effect of always quieting and easing the patient. When the *forced respirations* were kept up for a short time, and the blood became surcharged with oxygen, it would be some time before attempts to breathe would be made—this was frequently observed; advantage was taken of this, and the blood surcharged with oxygen when the change from the cumbersome tracheal tube of the respiratory apparatus was made for an ordinary tracheotomy tube, when the patient was allowed to breathe for himself.

No mention has been made of the difficulty encountered in the operation after the patient revived and began to move uneasily about; these movements loosened the tube in the trachea and started hemorrhage, and as at this time the patient was depending upon the forced respiration for his life, the result was made uncertain. This was the most serious time in the operation. In the house were boarding three soldiers, William H. Lees, C. H. Eddy and Daniel Stuber, of the U. S. recruiting service; they were quickly summoned, and performed efficient service in restraining the patient. At this time, and before the tracheal tube was first inserted, considerable blood passed into the lungs; it was subsequently coughed out at the valve of the apparatus.

At twelve o'clock, mid-day, after the forced respirations had been under way *two and one-half hours*, an ordinary tracheotomy tube, as stated, was substituted for the tube of the apparatus, and the patient allowed to breathe for himself.

### QUANTITY OF MORPHINE TAKEN.

The amount of morphine taken by Mr. Burns was ascertained to be nearly twenty grains. He stated, on questioning, that he had a powder two inches long, three-fourths of an inch wide and about one-fourth of an inch thick, and that he took the half of it.

### ADVISABILITY OF ADMINISTERING PURE OXYGEN.

It has been suggested that pure oxygen might be better than ordinary air in artificial or forced respiration. My opinion is that it would be superfluous, under the conditions existing, when it would be necessary to resort to it; we have the *reduced hæmoglobine* calling for oxygen, and ready to grasp it in whatever form it is offered, and the supplying of pure oxygen would, on this account, appear superfluous, and unnecessarily complicate the apparatus.

### DELIRIUM TREMENS COMPLICATION.

To add to the uncertainties of the case, the night following the operation, a bad attack of delirium tremens set in, the patient became quite violent, requiring at times four or five men to restrain him. This condition passed away, and with the exception of abnormalities in the number of respirations, nothing further happened to complicate the case.

### OBSERVATIONS ON THE RESPIRATIONS.

In the variation in the number of respirations, we find one of the most interesting and instructive features of the whole case. When the operation was begun at nine o'clock, Saturday morning, the rate was *less* than *one* per minute; when completed three hours later, some *eighteen* or *twenty* per minute, and remaining at the normal number during the rest of the day. During the following night, they were again modified by the delirium, and the next day, or about twenty-four hours after the operation, had lowered to the unpleasant rate of *only six per minute*. Here they remained for a short time, then gradually came up to eight, ten, twelve and fifteen, and, two days after the operation, became normal in number and action. This remarkable variation can most easily be explained by assuming that the shock and paralysis to the system had been almost completely overcome by the forced respiration; that the effect of the residual poison in the blood did not exert its influence under the forced respirations, but that in the following twenty-four hours, under the ordinary conditions of the system, the poison *remaining* again produced its effects; and had not the eliminative organs steadily continued to rid the system of the poison, secondary poisoning might have taken place, with serious, if not fatal, results.

From these conditions, a two-fold deduction may be made:

First—That some narcotic poisons, even if absorbed in sufficient quantity to produce fatal results, may, by *forced artificial respiration*, be prevented from exercising their influence on the organism.*

Second—That an instrument intended for the production of *forced respiration* should be so arranged that the respirations may be readily renewed within twenty-four hours.

ELIMINATION OF THE POISON.

The catheter was used three or four times daily for the two days succeeding the operation, after which, the paralyzation of the bladder being overcome, it was discontinued. Owing to the cathartics administered early in the case, followed by others after the patient had been resuscitated, good movements of the bowels came on early, with good results.

APPARATUS FOR FORCED RESPIRATION.

With these general remarks upon this case, which in many respects I believe to be one of the most interesting recorded, I will call your attention to the essential conditions required in an instrument adapted to the performance of forced respiration:

First—An air-forcing apparatus—ordinary bellows.

Second—A tracheal tube, which may be left in the trachea for twenty-four hours or more if required, and fitted with annular corrugations or a rubber tampon, as in the rubber tampon of Semen, to permit it to be securely held or tied in the trachea.

Third—A valve so arranged that the surgeon can completely control the passage of the air from the bellows to the lungs, and, when free, will permit the air to pass readily out and into the lungs, or, in other words, so that the surgeon need only manipulate the valve during the inspirations.

Fourth—To prevent the movements of the patient from disarranging the tube in the trachea, a flexible rubber tube connection should be made between the tracheal tube and the air-

* If this should prove true in other cases, it would almost imply that death need not result from opium poisoning when forced respiration is employed. The facts in this case are just as stated.

supply valve; and to insure greater convenience in manipulation, a flexible connection should be provided between the air-supply valve and the bellows-apparatus.

Fifth—The tracheotomy tube should be supplied with a movable piece, to which the rubber tubing can readily be attached or detached, to allow the respirations to be renewed when required.

With an apparatus of this form, simple in its working, practical, reasonable in expense, and supplemented by foot-bellows, I believe it possible for a practitioner, without an assistant, to make the entire operation and resuscitate a patient in as perilous a condition as our Mr. Burns was found to be.

An important addition to this apparatus, which could easily be added and not complicate the operation, would be a vessel of tin, or copper, arranged similar to a Woolf bottle, in which the air passing through water, heated with a spirit lamp, and medicated, if desired, with a suitable stimulant, could be used in cold weather. The value and simplicity of such an apparatus, supplied with a thermometer to gauge the temperature of the water or air, is at once apparent; the depression produced by forcing *cold* air into the lungs could be obviated, and consequent injury to the lung tissue would be reduced to a minimum.

In this small satchel, I have the first complete apparatus of this kind (excepting the vessel for heating the air); its portability is apparent, and for *forced respiration* on man, or in the physiological laboratory, its convenience and practicability would appear unquestioned.

### WILL FORCED RESPIRATION INJURE THE LUNGS?

In the case of Mr. Burns, not the slightest evidence was given of any injury to the lung tissue from the forced respirations; the judgment of the surgeon must be exercised here and prevent over-inflation of the lungs; with a valve in the apparatus to control the inspirations as previously described, there need be no apprehension on this point.

FORCED RESPIRATION WITH INTUBATION OF THE LARYNX.

The application of *intubation* of the larynx to forced respiration, I have considered, and while believing it may yet be applied, see many difficulties which may possibly be overcome, but even then do not consider it will offer such positive results as the performance of tracheotomy. The simple apparatus I have supplied could be used as readily in controlling the respirations in intubation as in tracheotomy.

With these remarks, gentlemen, I leave the subject for your consideration, adding only that forced respiration may be made available, not only in opium poisoning, but also in almost all varieties of conditions in which artificial respiration has been used, and with the certainty that it will be a more potent means of saving life than the latter.

Furthermore, its possibilities can only be determined by actual experiment in many cases where any other means at our commands must certainly fail.

